# The Effect of Exposure to Candida Albicans Suspension on the Properties of Silicone Dental Soft Lining Material

**DOI:** 10.3390/ma17030723

**Published:** 2024-02-02

**Authors:** Grzegorz Chladek, Michał Nowak, Wojciech Pakieła, Izabela Barszczewska-Rybarek, Jarosław Żmudzki, Anna Mertas

**Affiliations:** 1 Materials Research Laboratory, Faculty of Mechanical Engineering, Silesian University of Technology, 18a Konarskiego Str., 41-100 Gliwice, Poland; 2 Nova Clinic, 22 Jankego Str., 40-612 Katowice, Poland; michalnowak83@yahoo.pl; 3 Department of Engineering Materials and Biomaterials, Faculty of Mechanical Engineering, Silesian University of Technology, 18a Konarskiego Str., 41-100 Gliwice, Poland; wojciech.pakiela@polsl.pl (W.P.); jaroslaw.zmudzki@polsl.pl (J.Ż.); 4 Department of Physical Chemistry and Technology of Polymers, Faculty of Chemistry, Silesian University of Technology, Strzody 9 Str., 44-100 Gliwice, Poland; izabela.barszczewska-rybarek@polsl.pl; 5 Department of Microbiology and Immunology, Faculty of Medical Sciences in Zabrze, Medical University of Silesia in Katowice, 19 Jordana Str., 41-808 Zabrze, Poland; amertas@sum.edu.pl

**Keywords:** silicone elastomers, denture soft linings, *Candida albicans*, mechanical properties, colonization, penetration, dentures

## Abstract

While functioning in the oral cavity, denture soft linings (SL) are exposed to contact with the microbiota. Dentures can offer perfect conditions for the multiplication of pathogenic yeast-like fungi, resulting in rapid colonisation of the surface of the materials used. In vitro experiments have also shown that yeast may penetrate SL. This may lead to changes in their initially beneficial functional properties. The aim of this work was to investigate the effect of three months of exposure to a *Candida albicans* suspension on the mechanical properties of SL material and its bond strength to the denture base polymer, and to additionally verify previous reports of penetration using a different methodology. Specimens of the SL material used were incubated for 30, 60 and 90 days in a suspension of *Candida albicans* strain (ATCC 10231). Their shore A hardness, tensile strength, and bond strength to acrylic resin were tested. The colonization of the surface and penetration on fractured specimens were analysed with scanning electron and inverted fluorescence microscopes. Exposure to yeast did not affect the mechanical properties. The surfaces of the samples were colonised, especially in crystallized structures of the medium; however, the penetration of hyphae and blastospores into the material was not observed.

## 1. Introduction

The problem of edentulism occurs with varying intensity depending on the age of patients and the wealth of societies. Peltzer et al. [1] reported that among adults older than 50 years of age, its overall prevalence was 21.7% in Mexico; 16.3% in India; 9% in China; and 8.5% in South Africa, while in the United States in patients over 15 years of age the prevalence of edentulism was 4.9% [2]. The basic method of rehabilitation for these patients is the use of dentures, which are usually made of polymer materials [3,4]. Soft denture polymeric relining materials are used in new and old dentures at the mucosal interface. They allow for a more even distribution of the chewing forces transferred to tissues, but also show a cushioning effect during chewing, helping to improve retention and stabilisation of dentures [5,6]. They are recommended mainly for patients with atrophied or acute alveolar ridges, painful mucosa under the denture or during treatment for mucosal healing caused by a hard denture, or after surgical procedures [7,8]. Studies show significant clinical benefits associated with the long-term use of mostly silicone soft linings, achieved by improving chewing ability [9,10,11] and other factors affecting the comfort of patients [12,13,14,15]. Among the problems related to the functioning of soft liners in clinical practice, their relatively quick colonisation by yeast-like fungi is indicated [16,17]. Research shows that after a year, approximately 30% of soft silicone relinings are colonised so intensively that they need to be replaced [18].The importance of the occurrence of pathogenic microflora in dentures applies not only to the well-known complications of the oral cavity [19,20]. The microorganisms of dentures can be both inhaled and swallowed, thereby affecting other aspects of the health of elderly patients, due the infection of the lungs, kidneys, cardiovascular, and digestive systems [21]. To eliminate complications, experimental long-term materials with antimicrobial properties have been considered [22,23,24,25,26,27], but none of them has been introduced into clinical practice so far. Much attention has also been paid to the possibility of disinfecting and cleaning soft liners, but its effectiveness is also limited [28,29], as it may negatively affect their physicochemical properties [30,31].

Numerous studies have also been focused on better understanding the colonisation process and its potential relation to the properties of materials. To date, it has not been possible to determine whether, and to what extent, roughness affects the colonisation of these types of materials, as some studies have indicated this relationship [32,33,34], while it was not observed in others [35]. This indicates that roughness may be one of the important factors for this process, but probably not the decisive one. In this context, it is also suggested that *C. albicans* show greater adherence to surfaces with higher hydrophilicity [35]. In vitro investigations also show that the presence of salivary film on silicone liners influences early colonisation of Candida by decreasing the number of adhered yeast cells [36], however, after a longer period, the effect may be the opposite [37]. In vitro studies indicated that yeasts not only colonise the surface of soft linings, but also penetrate their interior [32,38,39]. During experiments, samples were exposed to suspensions of *C. albicans*, then cut and evaluated in microscopic observations. Bulad et al. [38] registered blastospores and hyphae forms inside soft acrylic and silicone linings, counted in hundreds or even thousands per analysed section. Moreover, in some sections, microorganism cells were observed at the interface where the denture base acrylic and the lining material were bonded, so the authors suggested that these data may indicate the influence of yeast presence on the properties of the bonded area. In laboratory studies, Rodger et al. [39] also indicated that just after 72 h of exposure to *C. albicans* suspension, the process of the penetration of yeasts into silicones occurs with considerable intensity. The presence of yeast inside the material may be an important factor influencing the mechanical properties of soft-lining silicones, but this problem has not been investigated so far. 

Thus, the purpose of the work was to investigate the mechanical properties of silicone soft lining material, and its bond strength with acrylic denture base polymer exposed to *C. albicans* suspension. Our hypothesis was that soft-lining samples exposed to a *C. albicans* strain suspension are colonised and penetrated by microorganism cells, which causes deterioration of mechanical properties. An additional objective was to investigate the penetration of *C. albicans* into silicone soft lining material using fractured samples during microscopic observations. Recognising the problems that are the subject of research is particularly important in the context of risk identification, control, and prevention of the spread of pathogenic microorganisms operating on dentures as a result of their contact with food ingredients and saliva.

## 2. Materials

### 2.1. Materials and Samples Preparation

The material tested was room-temperature silicone vulcanizing soft lining material Mollosil Plus, with a dedicated adhesive (Detax GmbH, Ettlingen, Germany). For the tensile bond strength test, shade 4—translucent Vertex Rapid Simplified acrylic heat-curing resin (Vertex-Dental B.V., Soesterberg, The Netherlands) was used as the denture base material. The materials were cured according to the manufacturer’s instructions.

Samples for Shore hardness and tensile tests were prepared in stainless steel moulds. After mixing the base and catalytic pastes at a 1:1 (mass) ratio, the material was put into the moulds using inserts made of 23 µm thick polyester film (DuPont Teijin Films, Chester, PA, USA), between the polymerized material and the lid of the mould, in order to prevent strong adhesion of the silicones to the smooth metal surface. The moulds were compressed using a hydraulic press (0.2 MPa) to remove air bubbles and excess material. For hardness tests, samples with a diameter of 40 mm and a height of 6 mm were made [40]. For tensile strength tests, firstly plates measuring 60 mm × 60 mm and 1.5 mm thick were polymerized, and then samples of type ISO 37-4 [41] were cut using the ZCP 020 cutting-die (Zwick GmbH & Com, Ulm, Germany).

Tensile bond strength samples were prepared based on the ISO 10139 standard [42], with necessary modifications aimed at the proper performance of the experiment. First, PMMA samples with dimensions of 20 × 20 mm and a thickness of 3.3 ± 0.1 mm were made with the standard flasking technique used in prosthetic dentistry. Their surfaces were individually wet ground (Labo-Pol25, Struers, Willich, Germany) with abrasive paper P500 (Struers, Willich, Germany), rinsed in distilled water, and conditioned in distilled water at 37 ± 1 °C for 2 days. 

**Figure 1:**
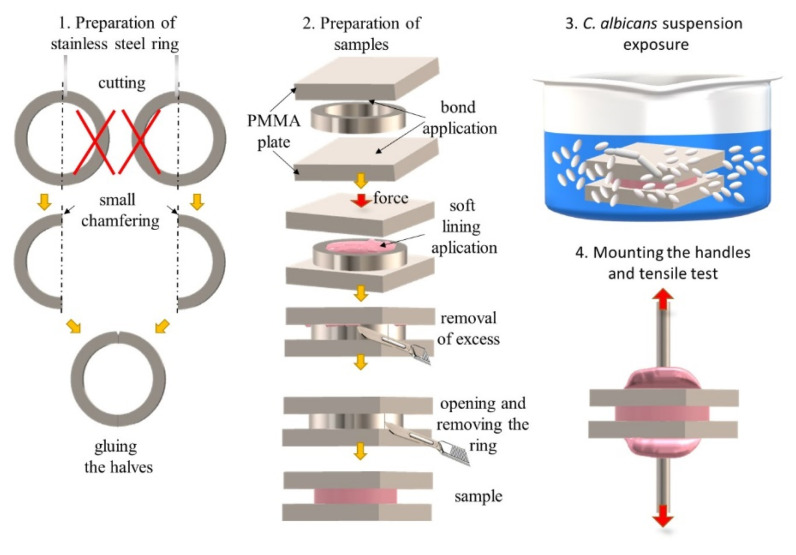
Illustration of the procedure for determining the impact of the presence of *C. albicans* on bond strength.

The samples were prepared according to the procedure shown in Figure 1. Stainless steel rings with an internal diameter of 10.8 mm, a height of 3 mm, and a wall thickness of 1 mm were prepared. The rings were made by gluing two halves together with Kropelka cyanopane glue (Feneedur, Montevideo, Uruguay). The halves of the rings were obtained by cutting two separate rings on a precision cutter so that, taking into account the thickness of the disc, two identical halves were obtained. A chamfer was made at one end of each half. The bonding agent was applied with a brush on the surface of the PMMA samples, the silicone soft lining material was placed into the ring set on an acrylic plate, and the second PMMA was pressed. After finishing the curing procedure, excess material was carefully removed with a scalpel, then the ring was opened and removed. This procedure was aimed at ensuring free access of the microorganism suspension to the surface of the samples during their incubation in the *C. albicans* suspension. After the exposure, handles (M4 screws) were attached to the samples using reducers placed in the jaws of a testing machine, and Vertex Castapress cold-curing acrylic resin (Vertex-Dental B.V., Soesterberg, The Netherlands) as previously described [23], and a tensile test was carried out.

### 2.2. Exposure to Suspension of Candida albicans

As the test medium, a suspension of the reference strain *C. albicans* (ATCC 10231) (CAS) in liquid Sabouraud medium diluted 5-fold with 0.9% NaCl solution (the final density was 3 × 10^6^ CFU/mL) was used. Sabouraud liquid medium (bioMérieux, Craponne, France) diluted 5 times with PBS was used as control medium (CM). All samples were plasma sterilized, and incubated for 30, 60 and 90 days at a temperature of 37 °C. The CM and TM were changed twice a week. During the media exchange, the samples were gently rinsed in CM.

### 2.3. Mechanical Properties Tests

For the tensile strength test, ten samples of each material were made for each experimental condition (*n* = 70). Specimens were mounted with polymeric tweezers in the jaws of a Zwick Z020 universal testing machine (Zwick GmbH & Com, Ulm, Germany). Tensile speed was 10 mm/min [43], and the tensile strength was calculated as a ratio of force at rupture (N) and initial cross-sectional area (mm^2^). Each fractured sample was carefully removed to a Petri dish with polymeric tweezers (parts were caught near the place of attachment in the jaw), and stored inside desiccators before microscopic investigations.

Ten samples of each material were made for the tensile bond strength (TBS) test for each experimental condition (*n* = 70). After each incubation time, samples were individually mounted in reducers placed in the jaws, and the tensile test was performed with a cross-head speed of 10 mm/min [23]. The tensile bond strength (TBS) was calculated as a ratio of the maximal registered force (N) and the initial cross-sectional area (mm^2^). During mounting/removal of the samples in/from reducers, special care was taken to touch only the handles, and fracture samples were collected on Petri dishes and stored inside desiccators before microscopic investigations. For each of the specimens, the type of failure was classified as follows: adhesive (debonding of the silicone material from the PMMA), cohesive (failure only within the lining material), and mixed (lining material partially left on the PMMA substrates).

Three samples were made for the Shore A hardness test for each incubation time (*n* = 21), and after 5 s of loading at five points of every sample [40], values were registered with a Bareiss HPE II-A durometer (Bareiss Prüfgerätebau GmbH, Oberdischingen, Germany). Since, due to the purpose of the experiment, it was not possible to carry out measurements on the same samples at subsequent time points, the hardness of all samples was measured before the start of incubation to confirm the lack of initial differences in hardness.

All tests were carried out in air-conditioned laboratory rooms at a temperature of ~21 °C.

### 2.4. Scanning Electron Microscopy Investigations

After exposure, samples were removed with tweezers and gently placed three times in ~30 mL of 4% glutaraldehyde solution (Pol-Aura, Zabrze, Poland) in a sterile 0.9% PBS solution, to remove loose and unattached cells, placed in the solution as above for 2 h to fix the cells [32,38], and then dehydrated in 100% alcohol [32] for one minute. For scanning electron microscopy (SEM) investigations, five parts of fractured specimens were chosen randomly after the tensile strength test, and five after the tensile bond strength test (ten parts for each incubation time). Due to the creation of a new surface after the rupture of the samples, the fixation was repeated as previously described, but the samples were dried (20–30 min) [44] at 30 °C in desiccators containing freshly dried silica gel. All samples were gold sputtered. A Zeiss SUPRA 35 scanning electron microscope (Zeiss, Oberkochen, Germany) was used for qualitative evaluation of surface colonisation and potential penetration (on fractured surfaces—interior of specimens) of *C. albicans*. The accelerating voltage was 15 kV.

### 2.5. Fluorescence Microscope Investigations

The five samples (tensile test samples) for each incubation time were carefully rinsed by gently dipping them three times in sterile PBS to remove loosely attached cells. Attached cells were fixed by immersion in 100% methanol for one minute and then air-dried [32]. After the tensile strength test, ten parts (five for surface observations and five for interior observations) were obtained. For surface observations 1–2 drops of Calcofluor White Stain (Sigma-Aldrich, St. Louis, MO, USA) were placed on a microscopy glass slide, the investigated surface was placed in it and after 1–2 min of incubation, observations were carried out at room temperature under UV light. For investigations of the interior (fractures), samples containing the tested surface (fracture) were carefully cut off from the fractured half-samples with a razor blade, approximately 3 mm from the observed surface. This was necessary due to the original size/geometry of the samples required to allow setting them correctly on a microscope slide for fluorescence microscopy. An amount of 1–2 drops of Calcofluor White Stain was placed on the glass slide, the investigated surface (fracture) was placed in it and after 1–2 min of incubation, observations were carried out at room temperature under UV light with fluorescence microscopy. Due to the binding of calcofluor with the cellulose and chitin contained in the cell walls of fungal microorganisms, they are fluorescent and visible as bright green to blue in UV-mode. During investigations an OLYMPUS IX 51 (Olympus, Tokyo, Japan) inverted fluorescence microscope was used for qualitative evaluation of surface colonisation and potential penetration of specimens.

### 2.6. Statistical Analysis

The results were analysed with PQStat ver. Software 1.6.6.204 (PQStat Software, Poznań, Poland). The distributions of the residuals were tested with the Shapiro–Wilk test (α = 0.05). The equality of variances was tested with the Levene test (α = 0.05). To compare Shore A hardness, tensile strength, and tensile bond strength before and after the experiment, a one-way ANOVA test was used (α = 0.05). If the null hypothesis was rejected, the Tukey’s HSD post hoc test (α = 0.05) was performed to find mean values that are significantly different from each other. For particular time intervals, CM vs. CAS values of hardness, tensile strength, and tensile bond strength were tested with Student’s *t*-test (α = 0.05).

## 3. Results

The results of the mechanical properties tests are summarized in Table 1. Shore A hardness ranged from 31.4 to 33.7 Shore A. Statistically significant changes after exposure to CM (*p* < 0.0001) and CAS (*p* < 0.0001) were recorded, however, an increase in hardness was only in comparison to values BE. There were no statistically significant differences in hardness between 30 and 90 days of the experiment (*p* > 0.05). The hardness values obtained after individual times did not differ statistically significantly after exposure to CM and CAS (*p* > 0.05).

Tensile strength ranged from 3.14 to 3.34 MPa. There were no statistically significant changes during exposure to CM and CAS (*p* > 0.05). For particular exposure times, there were no statistically significant differences (*p* > 0.05) after exposure to different media. 

Tensile bond strength ranged from 1.42 to 1.61 MPa. There were no statistically significant changes in its values with prolonged incubation time for both media (*p* > 0.05), as well as for particular exposure times, and no statistically significant differences after exposure to CM and CAS (*p* > 0.05). Failures of all registers were classified as cohesive type. 

**Figure 2:**
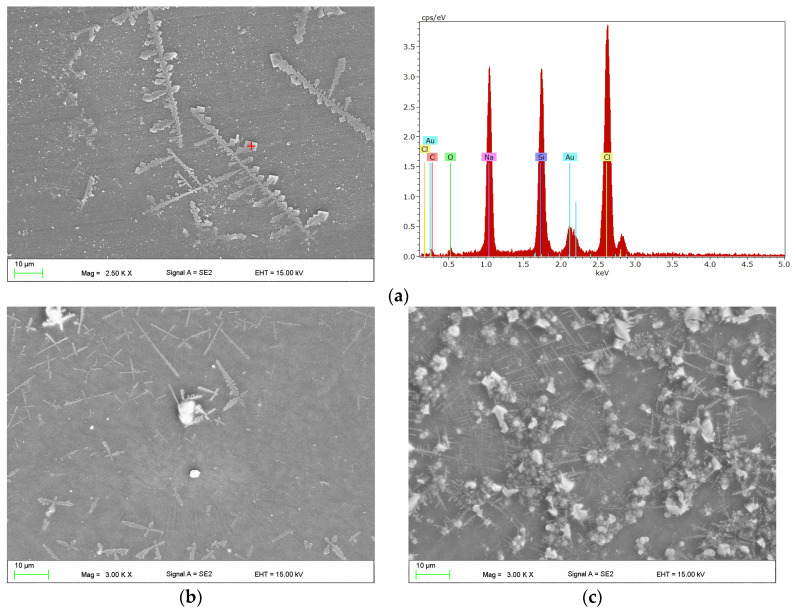
Scanning electron microscopy (SEM) micrographs of the surface of samples after 30 days (**a**,**b**); with exemplary dispersive X-ray spectroscopy (EDS) spectra (**a**); and after 90 days (**c**) of incubation in control medium (CM).

Examples of SEM micrographs showing the surfaces of samples after exposure to CM are presented in Figure 2. The presence of crystallized structures of the CM substrate were observed. The EDS analysis confirmed that they were composed of sodium and chlorine, so can be identified as crystallized NaCl from the PBS (Figure 2a). Typically, their quantity in the observation fields increased with increasing exposure time (please compare Figure 2a,b to Figure 2c), however areas with a large quantity for the shortest incubation time, and with a small number after 90 days, were also registered.

**Figure 3:**
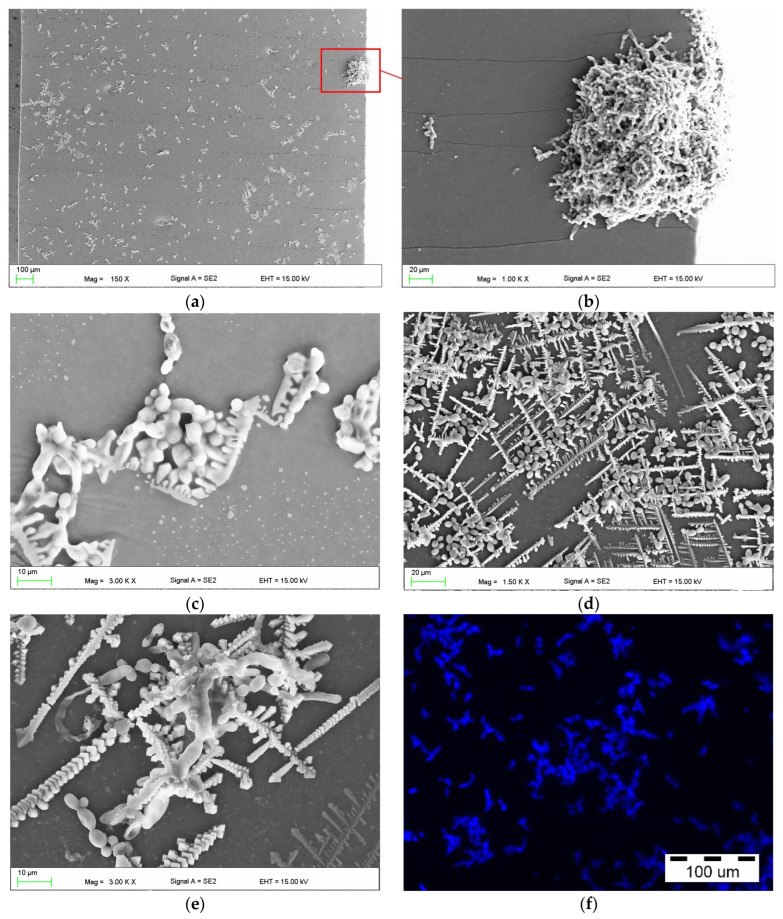
Representative micrographs of adhered *C. albicans* cells on the surface of samples incubated in CAS after tensile tests: SEM microscope after 30 days (**a**–**c**) and 90 days (**d**,**e**), and inverted fluorescence microscope after 30 days (**f**).

**Figure 4:**
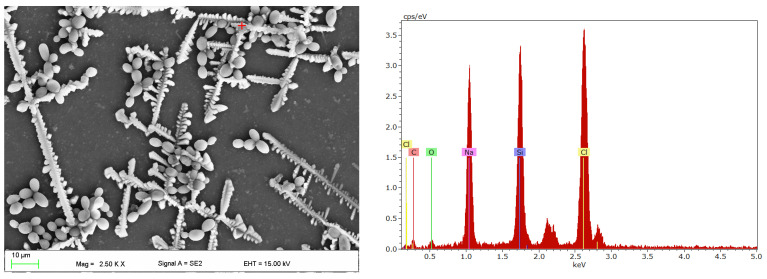
SEM micrographs of the surface of the sample after 90 days of incubation in *C. albicans* suspension with EDS spectrum of crystallised NaCl.

After incubation in CAS, the yeast cells were observed on surfaces using a SEM microscope and a fluorescence microscope (Figure 3). Numerous blastospores, pseudohyphae and, much less often, the presence of hyphae, were observed. SEM studies showed that the presence of fungal colonies was associated with the presence on the surface of crystallised structures composed of sodium and chlorine (Figure 4), that can be identified as NaCl from the PBS used in the medium. The cells were located between the elements of these structures, as shown in Figure 3e,f. On four samples only, very large colonies such as those presented in Figure 3b,c, were observed in single locations, and their occurrence was not associated with longer conditioning times (two were observed after 30 d and the next two after 90 d). The presence of yeast colonies, which would occur without the presence of crystallised structures, was not noted.

**Figure 5:**
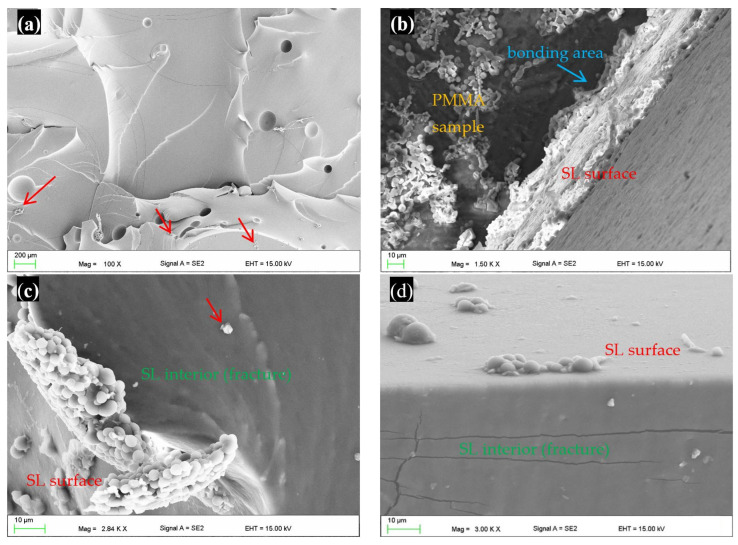

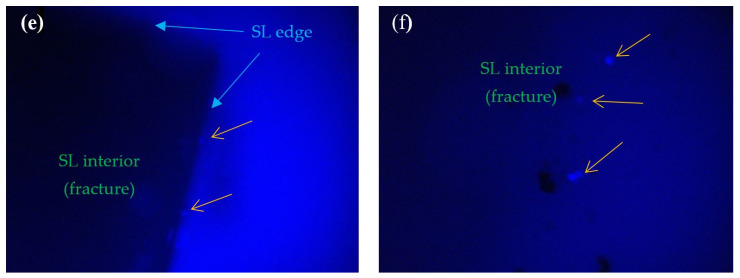
Micrographs of samples after the bond strength test showing the fracture after exposure to CM (**a**); surface/interior after incubation for 90 days in CAS and fractures (interior) after the tensile bond strength (**b**,**c**); and tensile strength tests after incubation for 90 days in CAS (**d**–**f**). SL—soft lining material.

The exemplary fracture of a sample exposed to CM after the bond strength test is presented in Figure 5a. Some bubbles measuring up to 200 mm were visible, which formed due to the mixing of the material components during polymerisation, as well as a small number of loosely bound particles indicated by red arrows, that were formed during the destruction of the samples. Representative SEM micrographs of samples incubated in CAS after the bond strength test are presented in Figure 5b,c. Numerous colonies of *C. albicans* were observed on the surface of the denture base PMMA material (Figure 5b). 

The presence of yeast colonies was also observed near the bonding zone (PMMA—bonding agent—SL) (Figure 5b). *C. albicans* was observed on the surface of the SL, and in some places it had formed a compact biofilm (Figure 5c). After the test, the presence of delaminated and cracked biofilm was also observed at the edges of the fractures, however this was rare (Figure 5c). The presence of *C. albicans* was not recorded on the surface of the fractures, but contaminations (material particles) were observed. 

Fracture analysis using SEM, obtained during the tensile strength, also showed no presence of *C. albicans* inside the samples, although colonies were visible on the surface near the edges (Figure 5d). Observations using a fluorescence microscope confirmed the presence of *C. albicans* on the surface of the samples near the edges (yellow arrows, Figure 5e). In the case of one of the samples, after 90 days of incubation in CAS, yeasts were observed on the surface of one fracture in two observation fields—in the first three blastospores (Figure 5f), and in the second four blastospores.

## 4. Discussion

*C. albicans* belongs to the commensal microflora of up to 65% of healthy individuals; however, its prevalence in denture wearers is much higher, increasing the risk of infection [45]. The growth of yeast-like fungi such as *C. albicans* is also a significant problem experienced after the application of soft linings [46]. Due to favourable environmental conditions (decreased flow of oxygen and saliva, and local acidity) [20], as well as objective difficulties in maintaining optimal hygiene of dentures and the original properties of materials, microorganisms can adhere to their surface within a short period, which creates the risk of negative consequences for patients’ health [47]. *C. albicans*, with other less widespread *Candida* species, have been well recognised for their role in denture stomatitis [48,49], a typical disorder that appears under dentures and is manifested by inflammation and erythema of the oral mucosal areas [21]. *C. albicans* can also be an opportunistic pathogen causing recurring mucosal infections as well as lethal invasion infections, especially if the patient is under an immunoincompetent and immunosuppressed condition [50]. Moreover, yeast cells in such a difficult environment can often survive antifungal therapy [20,51]. *Candida* species can cause tissue irritations by releasing metabolic substances [52]; in the case of candidiasis, they can cause symptoms such as burning, painful sensations, and taste disorders [53]. The oral cavity may also colonize the upper gastrointestinal tract and respiratory system [54,55]. Due to these problems, microbiological colonisation is mentioned as one of the major reasons for relatively rapid replacement of soft denture liners in clinical practice.

Although relatively more attention has been paid to surface colonization of soft dental lining materials by microorganisms and their consequences, published reports on the penetration of *C. albicans* into their interior are very rare. Despite the importance of the problem, only a few in vitro studies and one in vivo study are available. Previous in vitro studies [32,38,39,56] have shown that *C. albicans* penetrated silicone soft lining materials in a short time. Burns et al. [56] were the first to investigate the possibility of yeast penetration into soft denture liners. Samples were incubated in a solution of Sabouraud agar with cultures of dextrose and *C. albicans*, changed weekly. After 8 weeks, the samples were sectioned using a microtome with water cooling, and evaluated under a microscope. A mean of 50 yeast cells per observation field (magnification ×400) were noted, and cells were observed in all cross-sections of silicone samples. Bulad et al. [38] used a similar methodology, and after exposure for 6 weeks in a suspension of *C. albicans* (in artificial saliva of a composition not specified), in cross sections (with dimensions of 1.5 × 10 mm) observed from over a thousand to almost four thousand blastospores per sample. The number of blastospores decreased with the distance from the surface of the samples. Furthermore, penetration of the hyphae forms was confirmed in all cross sections (deeper regions were less penetrated), and at the junction of the denture base acrylic with the soft lining. The authors suggested that this last finding indicated the influence of *C. albicans* penetration on the strength of the acrylic-lining bonding. Several years later, similar research showed that even after 72 h of incubation, thousands of blastospores and hyphae forms on 2 × 10 mm fields penetrated into silicone lining samples [39]. Krishnamurthy et al. [32], in an experiment very similar in terms of methodology to that presented in previously discussed works [38,39,56], independently confirmed the penetration of *C. albicans* blastospores and hyphae even into the deepest sections of different soft linings and bonding areas, and also suggested potential consequences for the mechanical properties. To date, only one study is available in which soft materials used in in vivo conditions have been tested, and this study did not confirm the penetration of yeast-like fungi into the interior [34]. Similar studies for other prosthetic materials are even more rare. An example is a work by Khiyani et al. [57], suggesting the possibility of penetration of yeast-like fungi into two denture base materials, but it only contains a very brief description of the research methodology. Another work indicates that *C. albicans* probably does not have the ability to penetrate into denture base materials [58]. The above-mentioned discrepancies and scarcity of data indicate the need for further research in this area.

Taking into account the afore-mentioned results, and also suggestions from Bulad et al. [38] and Krishnamurthy et al. [32], starting our experiment we expected significant changes in mechanical properties. Despite this, our tests did not show changes in the tensile strength or the tensile bond strength due to the presence of CAS. Only an increase in hardness was observed in the initial phase of the experiment, which was similar in CM and CAS, so it is related to the continuation of the crosslinking process at elevated temperature [8,59]. The obtained values of mechanical properties, as well as the dynamics of their changes over time, were comparable to the ones registered in other studies for silicone soft linings stored at elevated temperature [60,61,62,63,64,65,66]. 

Taking into account previous reports on *C. albicans* in the junction zone [32,38], during our experiment, particular attention was paid to the results of tensile bond strength results. The test samples were designed to not restrict the access of yeast cells to this area. The deterioration of this property was especially expected due to the fact that in clinical conditions, deboning is a frequent cause of problems related to the functioning of silicone soft linings [18]. However, in our tests these values were stable, and there was no change in the failure type (all was cohesive) after 90 days, which allows us to conclude that even if some changes occurred in the connection zone, its strength was still higher than that of the soft lining material [67,68].

Microscopic observations confirmed the presence of a large number of blastospores and pseudohyphae on the surface, but the form of the hyphae was only occasionally registered, so the distribution of the morphological forms was comparable to the ones registered by Burns et al. [56], where a similar CAS was used. In the three remaining experiments [32,38,39], more hyphae were observed; however, the exact composition of the CAS was not specified, so it is hard to judge how this could have affected the results of the experiment. 

In the interior of the material only seven blastospores at a single fracture were observed. It can be assumed that their presence was related to accidental contamination associated with the movement of samples immediately after their destruction on the testing machine, rather than the actual penetration of *C. albicans* into the material. This is supported by microscopic observations that showed fragments of biofilms detaching from the edges of the sample (Figure 5c). Our results are different from previous works that used samples cut into sections with a microtome in an aqueous environment, where the numbers of cells observed in the interior were very high, even counted in thousands, regardless of whether only blastospores, or blastospores and hyphae forms, occurred [32,38,39,56]. Therefore, the question arises about the potential risk of the cutting methodology influencing results, because the mentioned works do not describe how methodologies were validated to eliminate the risk of transfer of microbiological material to the interior during this process. It seems that the possibility of obtaining a false positive result was minimised to a greater extent in the current experiment. On the other hand, it cannot be ruled out that the fractures in our experiment were obtained in nonrepresentative places, because the destruction process is beyond the control of the testing machine operator; however, this seems unlikely, considering the number of fractures tested. It should also be noted that the cited works [32,38,39,56] did not discuss how *C. albicans* cells could penetrate various materials. Very strong penetration was observed after only 72 h of the experiment, which is particularly surprising when only blastospores were observed on the surface and inside [39]. It is difficult to explain how blastospores, which do not have the ability to actively move, penetrated the polymeric materials in such a short time, especially if we consider that the denture soft lining materials are not porous. It should be emphasised that our results are consistent with the only experiment in which soft lining materials used in clinical conditions were tested. Taylor et al. [34] observed a varied intensity of surface colonisation of soft linings silicones, but after six months of functioning in the oral cavity, penetration was not registered.

The obtained results indicate that the issue of potential penetration of *C. albicans* remains open and requires comprehensive laboratory tests. It seems very important to investigate microenvironmental factors in the context of the presence of different forms of candida, because numerous parameters such as pH values; carbonates/peptides presence and concentration; and temperature or initial concentration of cells in suspension, may influence the germ-tube formation process [69,70,71]. Nadeem et al. [72] reported that incubation temperature and pH value are especially significant for yeast or hyphal form creation. An incubation temperature of 37 °C favoured high germ tube formation, while 34 °C allowed low filamentation. A pH near 5 induced low filamentation, while a pH of 7.4 gave ideal conditions for germ tube induction. In our experiment, the temperature was maintained at 37 °C and the initial pH value was 6.7, which according to Nadeem et al. [72], should favour medium filamentation. The initial pH values of the culture medium used in our experiment also corresponded to the saliva recorded, which is normally ranged from 5.6 to 7.1, including for denture wearers [73,74]. However, the presence of Candida species promotes the lowering of pH values by producing cetate; pyruvate; formate; and propionate acids, which influence in vitro tests and under clinical conditions may activate candidal proteases; phospholipases; and collagenases, promoting tissue damage and subsequent invasion by yeast-like fungi [75,76]. The possibility of the pH values of the culture medium used in studies influencing the penetration of *C. albicans* into soft lining remains unknown. Thus, it is difficult to consider if they could have a significant impact on the results of the experiments; this relationship should be analysed in future.

The observed tendency for the occurrence of *C. albicans* colonies/cells in the PMMA-silicone junction zones characterised by the presence of micro-gaps, or within crystallized structures coming from components of the culture medium, is supported by previous experiments, where stronger colonisation of more rough surfaces was reported [32,33,77,78]. Therefore, the current results indicating the lack of penetration may indicate that problems with the increasing presence of microorganisms on soft lining materials under clinical conditions over time [79] are not caused by the penetration of yeasts, but by the formation of micro-damages on surfaces that are difficult for cleaning agents to access during long-term use. This supposition is supported by in vitro investigations, where greater surface roughness increased resistance to biofilm removal, and the number of hyphae and blastospores remaining on the surfaces of prosthetic polymers after the cleaning process [80]. 

This problem is particularly important considering that some methods of cleaning soft lining surfaces increase their roughness [81,82,83]. Moreover, a longer period of denture use also results in rougher surfaces of liners [84].

This study has some potential limitations. In the presented research study, only one set of conditions of *C. albicans* incubation was used. Due to the conditions under dentures, we need further experiments taking into account environmental conditions, such as different temperature values and pH affecting the germ-tube formation process. Another limitation was that a basic suspension of *C. albicans* was used, so future experiments after biofilm formation, including the possibility of synergistic interactions between various microorganisms [16,85,86], will pose a serious challenge. Attention should be paid to the lack of prior research studies on the topic, and objective methodological difficulties that could potentially influence the results of microscopic studies. Despite every effort, the risk of the influence of dynamic movement of samples after their rupture on the presence of microbial cells at the fracture surfaces, cannot be completely ruled out—cells could fall from the surface or contaminate it.

## 5. Conclusions

The current research did not show any effect of the presence of *C. albicans* suspension on the mechanical properties of denture soft lining material. Previous reports on the penetration of hyphae and blastospores into the material have also not been confirmed. Further, comprehensive research is necessary to help confirm or deny the possibility of Candida strains penetrating materials, including in vivo tests to link germ tube formation conditions, such as variable pH and temperature, with the possibility of colonising the surface and potentially also the interior of materials.

## Figures and Tables

**Table 1:** The results of Shore A hardness, tensile strength, and tensile bond strength investigations (average values and standard deviations) *.

Time, Days	Shore A Hardness, Shore A		Tensile Bond Strength, MPa		Tensile Strength, MPa	
	CM	CAS	CM	CAS	CM	CAS
BE	31.4 ± 0.9 ^A^	31.4 ± 0.9 ^A^	1.43 ± 0.15	1.43 ± 0.15	3.14 ± 0.5	3.14 ± 0.5
30	33.1 ± 0.6 ^B^	33.6 ± 0.9 ^B^	1.56 ± 0.10	1.51 ± 0.12	3.15 ± 0.37	3.23 ± 0.44
60	33.0 ± 1.1 ^B^	33.7 ± 0.9 ^B^	1.42 ± 0.17	1.61 ± 0.25	3.34 ± 0.40	3.29 ± 0.41
90	33.5 ± 0.6 ^B^	33.2 ± 0.9 ^B^	1.50 ± 0.10	1.41 ± 0.15	3.2 ± 0.58	3.15 ± 0.52

BE—before exposure, CM—control medium, CAS—C. albicans suspension. * Different uppercase letters (A,B) for columns show significantly different results at the level of *p* < 0.05.

## Data Availability

The datasets used and/or analysed during the current study are available from the corresponding author on reasonable request.

## References

[1] Peltzer K., Hewlett S., Yawson A.E., Moynihan P., Preet R., Wu F., Guo G., Arokiasamy P., Snodgrass J.J., Chatterji S. (2014). Prevalence of Loss of All Teeth (Edentulism) and Associated Factors in Older Adults in China, Ghana, India, Mexico, Russia and South Africa. Int. J. Environ. Res. Public Health.

[2] Slade G.D., Akinkugbe A.A., Sanders A.E. (2014). Projections of U.S. Edentulism Prevalence Following 5 Decades of Decline. J. Dent. Res..

[3] Bukleta M.S., Bukleta D., Selmani M., Kuhar M. (2019). Frequency of Complete and Removable Partial Denture Treatment in the Primary Health Centres in Three Different Regions of Kosovo from 2002 to 2013. Slov. J. Public Health.

[4] Ni S.C., Thomas C., Yonezawa Y., Hojo Y., Nakamura T., Kobayashi K., Sato H., Da Silva J.D., Kobayashi T., Ishikawa-Nagai S. (2022). Comprehensive Assessment of the Universal Healthcare System in Dentistry Japan: A Retrospective Observational Study. Healthcare.

[5] Braden M., Wright P.S., Parker S. (1995). Soft Lining Materials—A Review. Eur. J. Prosthodont. Restor. Dent..

[6] Shen C., Rawls H.R., Esquivel-Upshaw J. (2021). Phillips’ Science of Dental Materials.

[7] ELsyad M.A., Shaheen N.H., Ashmawy T.M. (2017). Long-Term Clinical and Prosthetic Outcomes of Soft Liner and Clip Attachments for Bar/Implant Overdentures: A Randomised Controlled Clinical Trial. J. Oral Rehabil..

[8] Kreve S., Dos Reis A.C. Denture Liners: A Systematic Review Relative to Adhesion and Mechanical Properties. https://www.hindawi.com/journals/tswj/2019/6913080/.

[9] Furuya Y., Kimoto S., Furuse N., Furokawa S., Igarashi K., Suzuki A., Kawai Y. (2022). Effectiveness of Silicone-Based Resilient Denture Liners on the Patient-Reported Chewing Ability: A Randomized Controlled Trial. J. Prosthodont. Res..

[10] Kar S., Tripathi A., Fatima T. (2019). A Comparative Study of Masticatory Performance in Complete Denture Patients before and after Application of Soft Liner. Med. J. Armed Forces India.

[11] Rastogi A., Srivastava S., Gaur A., Dupare A., Rastogi S., Kamatagi L. (2015). Electromyographic Evaluation of the Effect of Lined Dentures on Masticatory Muscle Activity in Edentulous Subjects. J. Clin. Diagn. Res..

[12] Mohsen Y.H., Kader M.A., Abdel Nabi N., Radi I.A.W. (2022). Satisfaction with Resilient Denture Liner versus Acrylic Resin Telescopic Prostheses for Patients with Ectodermal Dysplasia: A Nonrandomized Crossover Clinical Trial. J. Prosthet. Dent..

[13] Sônego M.V., Neto C.L.M.M., Dos Santos D.M., de Melo Moreno A.L., de M. Bertoz A.P., Goiato M.C. (2022). Quality of Life, Satisfaction, Occlusal Force, and Halitosis after Direct and Indirect Relining of Inferior Complete Dentures. Eur. J. Dent..

[14] Alqutaibi A.Y., Alnazzawi A.A., Farghal A.E., Bakr R.M., Mahmoud I.I. (2023). Impact of Acrylic and Silicone-Based Soft-Liner Materials on Biting Force and Quality of Life of the Complete Denture Wearers: A Randomized Clinical Trial. J. Clin. Med..

[15] Shinde J., Mowade T., Gupta P., Tekale R., Pande N., Deshmukh K., Lokhande T., Radke U. (2022). Satisfaction in Conventional Acrylic Complete Denture Patient with and without Denture Liners—A Systematic Review. Pan. Afr. Med. J..

[16] Valentini F., Luz M.S., Boscato N., Pereira-Cenci T. (2013). Biofilm Formation on Denture Liners in a Randomised Controlled in Situ Trial. J. Dent..

[17] Tasopoulos T., Vrioni G., Naka O., Diamantatou T., Zoidis P., Tsakris A. (2022). Adherence of Candida Albicans to Five Long-Term Silicone-Based Denture Lining Materials Bonded to CAD-CAM Denture Base. J. Prosthodont..

[18] Mutluay M.M., Oguz S., Fløystrand F., Saxegaard E., Dogan A., Bek B., Ruyter I.E. (2008). A Prospective Study on the Clinical Performance of Polysiloxane Soft Liners: One-Year Results. Dent. Mater. J..

[19] Smith A., Williams D., Bradshaw D., Milward P., Kutubi S.A., Rowe W. (2020). The Effect of Residual Food Stain on Candida Albicans Colonisation of Denture Acrylics. Dent. Oral Biol. Craniofacial Res..

[20] Gleiznys A., Zdanavičienė E., Žilinskas J. (2015). Candida Albicans Importance to Denture Wearers. A Literature Review. Stomatologija.

[21] Gendreau L., Loewy Z.G. (2011). Epidemiology and Etiology of Denture Stomatitis. J. Prosthodont..

[22] Habibzadeh S., Eskandarion S., Marashi S.M.A., Yunesi G., Kharazifard M. (2021). Antifungal Efficacy of a Permanent Silicon Soft Liner Containing Silver Nanoparticles. Front. Dent..

[23] Jabłońska-Stencel E., Pakieła W., Mertas A., Bobela E., Kasperski J., Chladek G. (2018). Effect of Silver-Emitting Filler on Antimicrobial and Mechanical Properties of Soft Denture Lining Material. Materials.

[24] Deng J., Ren L., Pan Y., Gao H., Meng X. (2021). Antifungal Property of Acrylic Denture Soft Liner Containing Silver Nanoparticles Synthesized in Situ. J. Dent..

[25] Ahmed A.Q., Al-Hmedat S.J.A.-Z., Hanweet D.M., Haider J. (2023). Assessing the Antifungal Activity of a Soft Denture Liner Loaded with Titanium Oxide Nanoparticles (TiO_2_ NPs). Dent. J..

[26] Ansarifard E., Zareshahrabadi Z., Sarafraz N., Zomorodian K. (2021). Evaluation of Antimicrobial and Antibiofilm Activities of Copper Oxide Nanoparticles within Soft Denture Liners against Oral Pathogens. Bioinorg. Chem. Appl..

[27] Songsang N., Anunmana C., Pudla M., Eiampongpaiboon T. (2022). Effects of Litsea Cubeba Essential Oil Incorporated into Denture Soft Lining Materials. Polymers.

[28] AlHamdan E.M. (2022). Soft Denture Liner and Microbial Disinfection with Contemporary and Conventional Agents. Photodiagn. Photodyn. Ther..

[29] Hahnel S., Rosentritt M., Bürgers R., Handel G., Lang R. (2012). Candida Albicans Biofilm Formation on Soft Denture Liners and Efficacy of Cleaning Protocols. Gerodontology.

[30] Mańka-Malara K., Trzaskowski M., Gawlak D. (2021). The Influence of Decontamination Procedures on the Surface of Two Polymeric Liners Used in Prosthodontics. Polymers.

[31] Salloum A.M. (2014). Effect of Aging on Bond Strength of Two Soft Lining Materials to a Denture Base Polymer. J. Indian Prosthodont. Soc..

[32] Krishnamurthy S., Hallikerimath R.B. (2016). An In-Vitro Evaluation of Retention, Colonization and Penetration of Commonly Used Denture Lining Materials By Candida Albicans. J. Clin. Diagn. Res..

[33] Tari B.F., Nalbant D., Dogruman Al F., Kustimur S. (2007). Surface Roughness and Adherence of Candida Albicans on Soft Lining Materials as Influenced by Accelerated Aging. J. Contemp. Dent. Pract..

[34] Taylor R.L., Bulad K., Verran J., McCord J.F. (2008). Colonization and Deterioration of Soft Denture Lining Materials in Vivo. Eur. J. Prosthodont. Restor. Dent..

[35] Kang S.-H., Lee H.-J., Hong S.-H., Kim K.-H., Kwon T.-Y. (2013). Influence of Surface Characteristics on the Adhesion of Candida Albicans to Various Denture Lining Materials. Acta Odontol. Scand..

[36] Mutluay M.M., Oğuz S., Ørstavik D., Fløystrand F., Doğan A., Söderling E., Närhi T., Olsen I. (2010). Experiments on in Vivo Biofilm Formation and in Vitro Adhesion of Candida Species on Polysiloxane Liners. Gerodontology.

[37] De Foggi C.C., Ayres M.S.B., Feltrin G.P., Jorge J.H., Machado A.L. (2018). Effect of Surface Characteristics of Soft Liners and Tissue Conditioners and Saliva on the Adhesion and Biofilm Formation. Am. J. Dent..

[38] Bulad K., Taylor R.L., Verran J., McCord J.F. (2004). Colonization and Penetration of Denture Soft Lining Materials by Candida Albicans. Dent. Mater..

[39] Rodger G., Taylor R.L., Pearson G.J., Verran J. (2010). In Vitro Colonization of an Experimental Silicone by Candida Albicans. J. Biomed. Mater. Res. Part B Appl. Biomater..

[40] (2010). Rubber, Vulcanized or Thermoplastic—Determination of Indentation Hardness—Part 1: Durometer Method (Shore Hardness).

[41] (2017). Rubber, Vulcanized or Thermoplastic—Determination of Tensile Stress-Strain Properties.

[42] (2016). Dentistry—Soft Lining Materials for Removable Dentures—Part 2: Materials for Long-Term Use..

[43] Shirkavand S., Moslehifard E. (2014). Effect of TiO_2_ Nanoparticles on Tensile Strength of Dental Acrylic Resins. J. Dent. Res. Dent. Clin. Dent. Prospect..

[44] Cavalcanti Y.W., Wilson M., Lewis M., Williams D., Senna P.M., Del-Bel-Cury A.A., da Silva W.J. (2016). Salivary Pellicles Equalise Surfaces’ Charges and Modulate the Virulence of Candida Albicans Biofilm. Arch. Oral Biol..

[45] Gacon I., Loster J.E., Wieczorek A. (2019). Relationship between Oral Hygiene and Fungal Growth in Patients: Users of an Acrylic Denture without Signs of Inflammatory Process. Clin. Interv. Aging.

[46] Hashem M.I. (2015). Advances in Soft Denture Liners: An Update. J. Contemp. Dent. Pract..

[47] Chladek G., Żmudzki J., Kasperski J. (2014). Long-Term Soft Denture Lining Materials. Materials.

[48] Abuhajar E., Ali K., Zulfiqar G., Al Ansari K., Raja H.Z., Bishti S., Anweigi L. (2023). Management of Chronic Atrophic Candidiasis (Denture Stomatitis)—A Narrative Review. Int. J. Environ. Res. Public Health.

[49] Pachava K.R., Nadendla L.K., Alluri L.S.C., Tahseen H., Sajja N.P. (2015). Invitro Antifungal Evaluation of Denture Soft Liner Incorporated with Tea Tree Oil: A New Therapeutic Approach Towards Denture Stomatitis. J. Clin. Diagn. Res..

[50] da Silva W.J., Seneviratne J., Samaranayake L.P., Del Bel Cury A.A. (2010). Bioactivity and Architecture of Candida Albicans Biofilms Developed on Poly(Methyl Methacrylate) Resin Surface. J. Biomed. Mater. Res. B Appl. Biomater..

[51] Faot F., Cavalcanti Y.W., de Mendonça e Bertolini M., de Rezende Pinto L., da Silva W.J., Cury A.A.D.B. (2014). Efficacy of Citric Acid Denture Cleanser on the Candida Albicans Biofilm Formed on Poly(Methyl Methacrylate): Effects on Residual Biofilm and Recolonization Process. BMC Oral Health.

[52] Altinci P., Mutluay M., Söderling E., Tezvergil-Mutluay A. (2018). Antimicrobial Efficacy and Mechanical Properties of BAC-Modified Hard and Soft Denture Liners. Odontology.

[53] Patel M. (2022). Oral Cavity and Candida Albicans: Colonisation to the Development of Infection. Pathogens.

[54] Uzel N.G., Teles F.R., Teles R.P., Song X.Q., Torresyap G., Socransky S.S., Haffajee A.D. (2011). Microbial Shifts during Dental Biofilm Re-Development in the Absence of Oral Hygiene in Periodontal Health and Disease. J. Clin. Periodontol..

[55] Evren B.A., Uludamar A., Işeri U., Ozkan Y.K. (2011). The Association between Socioeconomic Status, Oral Hygiene Practice, Denture Stomatitis and Oral Status in Elderly People Living Different Residential Homes. Arch. Gerontol. Geriatr..

[56] Burns D.R., Burns D.A., DiPietro G.J., Gregory R.L. (1987). Response of Processed Resilient Denture Liners to Candida Albicans. J. Prosthet. Dent..

[57] Khiyani S., Mahajan A.B., Sarda A., Srivastava H., Sanap A., Bhalerao N.N. (2022). Comparison of Penetration of Candida Albicans in Two Denture Base Materials. J. Res. Adv. Dent..

[58] Chladek G., Nowak M., Pakieła W., Mertas A. (2022). Effect of Candida Albicans Suspension on the Mechanical Properties of Denture Base Acrylic Resin. Materials.

[59] Mancuso D.N., Goiato M.C., Zuccolotti B.C.R., Moreno A., dos Santos D.M., Pesqueira A.A. (2012). Effect of Thermocycling on Hardness, Absorption, Solubility and Colour Change of Soft Liners. Gerodontology.

[60] Mese A., Guzel K.G. (2008). Effect of Storage Duration on the Hardness and Tensile Bond Strength of Silicone- and Acrylic Resin-Based Resilient Denture Liners to a Processed Denture Base Acrylic Resin. J. Prosthet. Dent..

[61] Białożyt-Bujak E., Wyszyńska M., Chladek G., Czelakowska A., Gala A., Orczykowska M., Białożyt A., Kasperski J., Skucha-Nowak M. (2021). Analysis of the Hardness of Soft Relining Materials for Removable Dentures. Int. J. Environ. Res. Public Health.

[62] Wyszyńska M., Białożyt-Bujak E., Chladek G., Czelakowska A., Rój R., Białożyt A., Gruca O., Nitsze-Wierzba M., Kasperski J., Skucha-Nowak M. (2021). Analysis of Changes in the Tensile Bond Strenght of Soft Relining Material with Acrylic Denture Material. Materials.

[63] Mutahar M., Al Ahmari N.M., Gadah T.S., Kariri M.A.M., Madkhli H.Y., Somaili D.M., Mobarki Y.M.Y., Darraj O.A., Halawi S.M., Al Moaleem M.M. (2023). Comparative Evaluation of Hardness and Energy Absorption of Some Commercially Available Chairside Silicone-Based Soft Denture Liners and a Heat-Cured Soft Denture Liner. Clin. Cosmet. Investig. Dent..

[64] Vuksic J., Pilipovic A., Poklepovic Pericic T., Kranjcic J. (2023). Tensile Bond Strength between Different Denture Base Materials and Soft Denture Liners. Materials.

[65] Panda S.K., Reddy N., Manual L., Krishna C., Jagadeesh K.N., Saidath K., Babaji P. (2021). An In Vitro Evaluation of Tensile Bond Strength of Soft Liners Bonded to Different Denture Base Resins. Ann. Afr. Med..

[66] Al Taweel S.M., Al-Otaibi H.N., Labban N., AlFouzan A., Shehri H.A. (2021). Soft Denture Liner Adhesion to Conventional and CAD/CAM Processed Poly(Methyl Methacrylate) Acrylic Denture Resins-An In-Vitro Study. Materials.

[67] Wemken G., Burkhardt F., Spies B.C., Kleinvogel L., Adali U., Sterzenbach G., Beuer F., Wesemann C. (2021). Bond Strength of Conventional, Subtractive, and Additive Manufactured Denture Bases to Soft and Hard Relining Materials. Dent. Mater..

[68] Mutluay M.M., Ruyter I.E. (2007). Evaluation of Bond Strength of Soft Relining Materials to Denture Base Polymers. Dent. Mater..

[69] Valentijn-Benz M., Nazmi K., Brand H.S., van’t Hof W., Veerman E.C.I. (2015). Growth of Candida Albicans in Human Saliva Is Supported by Low-Molecular-Mass Compounds. FEMS Yeast Res..

[70] Pollack J.H., Hashimoto T. (1987). The Role of Glucose in the pH Regulation of Germ-Tube Formation in Candida Albicans. J. Gen. Microbiol..

[71] Thompson D.S., Carlisle P.L., Kadosh D. (2011). Coevolution of Morphology and Virulence in Candida Species. Eukaryot. Cell.

[72] Nadeem S.G., Shafiq A., Hakim S.T., Anjum Y., Kazm S.U. (2013). Effect of Growth Media, pH and Temperature on Yeast to Hyphal Transition in Candida Albicans. Open J. Med. Microbiol..

[73] Nikolopoulou F., Tzortzopoulou E. (2007). Salivary pH in Edentulous Patients Before and After Wearing Conventional Dentures and Implant Overdentures: A Clinical Study. Implant. Dent..

[74] Alshahrani F.A., AlToraibily F., Alzaid M., Mahrous A.A., Al Ghamdi M.A., Gad M.M. (2022). An Updated Review of Salivary pH Effects on Polymethyl Methacrylate (PMMA)-Based Removable Dental Prostheses. Polymers.

[75] Ramos-Pardo A., Castro-Álvarez R., Quindós G., Eraso E., Sevillano E., Kaberdin V.R. (2023). Assessing pH-dependent Activities of Virulence Factors Secreted by Candida Albicans. Microbiologyopen.

[76] Nikawa H., Hamada T., Yamamoto T. (1998). Denture Plaque—Past and Recent Concerns. J. Dent..

[77] Gedik H., Ozkan Y.K. (2009). The Effect of Surface Roughness of Silicone-Based Resilient Liner Materials on the Adherence of Candida Albicans and Inhibition of Candida Albicans with Different Disinfectants. Oral Health Prev. Dent..

[78] Nevzatoğlu E.U., Ozcan M., Kulak-Ozkan Y., Kadir T. (2007). Adherence of Candida Albicans to Denture Base Acrylics and Silicone-Based Resilient Liner Materials with Different Surface Finishes. Clin. Oral Investig..

[79] Ahmed N., Bhandari A., Sachdev A., Khan F., Malhotra S., Bashir T. (2015). A Clinical Study of Adherence of Candida Albicans to an Improved NBR Denture Soft Lining Material. J. Health Allied Sci. NU.

[80] Jackson S., Coulthwaite L., Loewy Z., Scallan A., Verran J. (2014). Biofilm Development by Blastospores and Hyphae of Candida Albicans on Abraded Denture Acrylic Resin Surfaces. J. Prosthet. Dent..

[81] Badaró M.M., Prates T.P., Leite-Fernandes V.M.F., de Cássia Oliveira V., de Freitas Oliveira Paranhos H., Silva-Lovato C.H. (2019). In Vitro Evaluation of Resilient Liner after Brushing with Conventional and Experimental Ricinus Communis-Based Dentifrices. J. Prosthodont..

[82] Saito T., Wada T., Kubo K., Ueda T., Sakurai K. (2020). Effect of Mechanical and Chemical Cleaning on Surface Roughness of Silicone Soft Relining Material. J. Prosthodont. Res..

[83] Ueda T., Kubo K., Saito T., Obata T., Wada T., Yanagisawa K., Sakurai K. (2018). Surface Morphology of Silicone Soft Relining Material after Mechanical and Chemical Cleaning. J. Prosthodont. Res..

[84] Valentini F., Luz M.S., Boscato N., Pereira-Cenci T. (2017). Surface Roughness Changes in Denture Liners in Denture Stomatitis Patients. Int. J. Prosthodont..

[85] Kim H.-E., Liu Y., Dhall A., Bawazir M., Koo H., Hwang G. (2020). Synergism of Streptococcus Mutans and Candida Albicans Reinforces Biofilm Maturation and Acidogenicity in Saliva: An In Vitro Study. Front. Cell Infect. Microbiol..

[86] Moraes G.S., Cachoeira V.S., Alves F.M.C., Kiratcz F., Albach T., Bueno M.G., Neppelenbroek K.H., Urban V.M. (2021). Is There an Optimal Method to Detach Candida Albicans Biofilm from Dental Materials?. J. Med. Microbiol..

